# Relative role of community transmission and campus contagion in driving the spread of SARS-CoV-2: Lessons from Princeton University

**DOI:** 10.1093/pnasnexus/pgad201

**Published:** 2023-07-03

**Authors:** Sang Woo Park, Irini Daskalaki, Robin M Izzo, Irina Aranovich, Aartjan J W te Velthuis, Daniel A Notterman, C Jessica E Metcalf, Bryan T Grenfell

**Affiliations:** Department of Ecology and Evolutionary Biology, Princeton University, Princeton, NJ 08544, USA; University Health Services, Princeton University, Princeton, NJ 08544, USA; Environmental Health and Safety, Princeton University, Princeton, NJ 08544, USA; Princeton University Clinical Laboratory, Princeton University, Princeton, NJ 08544, USA; Department of Molecular Biology, Princeton University, Princeton, NJ 08544, USA; Department of Molecular Biology, Princeton University, Princeton, NJ 08544, USA; Department of Ecology and Evolutionary Biology, Princeton University, Princeton, NJ 08544, USA; Princeton School of Public and International Affairs, Princeton University, Princeton, NJ 08544, USA; Department of Ecology and Evolutionary Biology, Princeton University, Princeton, NJ 08544, USA; Princeton School of Public and International Affairs, Princeton University, Princeton, NJ 08544, USA

## Abstract

Mathematical models have played a crucial role in exploring and guiding pandemic responses. University campuses present a particularly well-documented case for institutional outbreaks, thereby providing a unique opportunity to understand detailed patterns of pathogen spread. Here, we present descriptive and modeling analyses of SARS-CoV-2 transmission on the Princeton University (PU) campus—this model was used throughout the pandemic to inform policy decisions and operational guidelines for the university campus. Epidemic patterns between the university campus and surrounding communities exhibit strong spatiotemporal correlations. Mathematical modeling analysis further suggests that the amount of on-campus transmission was likely limited during much of the wider pandemic until the end of 2021. Finally, we find that a superspreading event likely played a major role in driving the Omicron variant outbreak on the PU campus during the spring semester of the 2021–2022 academic year. Despite large numbers of cases on campus in this period, case levels in surrounding communities remained low, suggesting that there was little spillover transmission from campus to the local community.

**Significance Statement**

University campuses present challenges to preventing SARS-CoV-2 transmission, due to a high proportion of asymptomatic infections and high contact rates. SARS-CoV-2 outbreaks on the Princeton University campus offer an unusually well-documented perspective, rooted in mass asymptomatic testing, further informed by mathematical modeling aimed at guiding policy decisions. Here, we show that this model can parsimoniously capture observed outbreak patterns on campus during different eras of control. Our modeling analysis further reveals that strong coupling between epidemic dynamics on campus and in local communities drives the early epidemic. Subsequently, especially in the Omicron era, superspreading events came to dominate transmission on campus, thereby weakening the dynamical coupling of campus and community outbreaks.

## Introduction

Predicting and controlling the spread of SARS-CoV-2 has remained a critical public health and scientific question throughout the ongoing SARS-CoV-2 pandemic (1). Rapid, asymptomatic transmission of SARS-CoV-2 has hindered intervention efforts, such as contact tracing (2). Social distancing measures have played major roles in preventing transmission, but can be difficult to maintain for a prolonged period (3). The development of vaccines has provided a safe means of reopening society, but uncertainty remains on their long-term effectiveness in preventing infection and transmission, especially in the face of new viral variants.

Mathematical models have played a significant role in guiding these pandemic responses and exploring control strategies (4–7). Models can help monitor key parameters that govern epidemic dynamics (8) and retrospectively estimate the impact of intervention measures in reducing transmission (9). These estimates can further inform projections of future scenarios and allow us to explore the endemicity of SARS-CoV-2 (10–13).

Mathematical models have also been widely deployed in planning campus reopenings. Researchers from various institutions in the US—including Cornell (14), Emory (15), Georgia Institute of Technology (16), and UC Berkeley (17)—modeled the feasibility of controlling the epidemic on their campuses and considered mass asymptomatic testing as their main intervention. Mathematical models also played crucial roles in helping to make decisions about reopening, ensuring that infrastructure (e.g. isolation methods and spaces, supporting isolation and quarantine with food, etc.) and levels of mitigation (masks, ventilation, testing frequency, etc.) were adequate and appropriate. These modeling efforts helped identify key parameters for control, such as the testing turnaround time, and provided support for implementing similar measures at other institutions. Coupling modeling efforts with real-life implementations in university campuses further provided unique opportunities to directly test model-based predictions of intervention effects in preventing the transmission of SARS-CoV-2 (14)—each university campus offers a relatively well-controlled epidemic setting with a relatively homogeneously behaving population (especially among undergraduate students). Campuses can also offer strong opportunities for control by nonpharmaceutical interventions, such as isolation and mask-wearing; mass asymptomatic testing further provides robust ascertainment for epidemic sizes, allowing for accurate understanding of epidemic patterns.

On the other hand, university campuses also present unique challenges to controlling an outbreak. A large fraction of asymptomatic infections (due to the young age of university students) and high-density interactions—such as eating in large dining halls and various social activities—can readily permit rapid transmission. These kinds of contacts are inherently difficult to keep track of, making contact tracing less effective. The impact of intervention measures is expected to vary across different university campuses, reflecting heterogeneity in campus settings such as compliance, resources, community prevalence, ventilation infrastructure, as well as effects of other interventions present on campuses. For example, Duke and Harvard Universities experienced moderate outbreaks at the beginning of the fall semester in 2021 when in-person classes were allowed, despite high vaccination rates and weekly asymptomatic testing protocols (18, 19), whereas the number of cases remained low in Princeton University (PU) during the same time period with similar levels of testing and vaccination. Here, we focused on the dynamics of SARS-CoV-2 on the PU campus alone to eliminate heterogeneities inherent to such comparisons; we return to comparisons with other campuses later in the discussion.

**Fig. 1.**
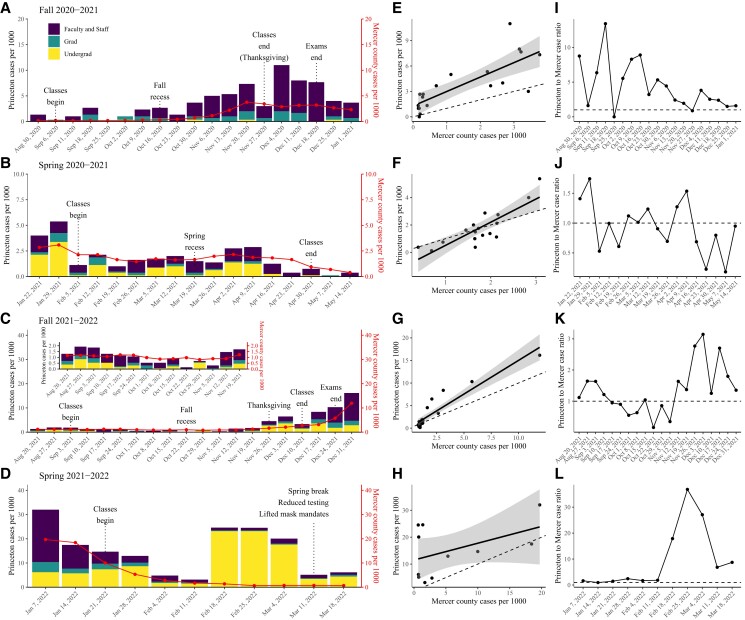
Dynamics of SARS-CoV-2 outbreaks in PU. A–D) Epidemic trajectories across four semesters: Fall 2020–2021 A), Spring 2020–2021 B), Fall 2021–2022 C), and Spring 2021–2022 D). Bar plots represent the weekly number of cases per 1,000 from both asymptomatic and symptomatic testing in PU. Solid lines represent the weekly number of cases per 1,000 in Mercer County. Number of cases in Mercer County is obtained from https://github.com/nytimes/covid-19-data. The weekly number of cases per 1000 in Princeton is normalized by the approximate total size of the PU population present on campus for each semester: 3,000 for Fall 2020–2021, 8,000 for Spring 2020–2021, 13,000 for Fall 2021–2022, and 15,000 for Spring 2021–2022. The weekly number of cases per 1,000 in Princeton in Mercer County is calculated based on the total population size as of 2020: 387,340 (www.census.gov). E–H) Correlations between the weekly number of cases per 1,000 in PU and in Mercer County. Solid lines and shaded areas represent the estimated linear regression lines and the associated 95% CIs. I–L) Ratios between weekly number of cases per 1,000 in PU and Mercer County. The dashed lines represent the 1:1 ratio.

We begin with a descriptive analysis of the PU outbreak (Fig. 1), then present modeling analyses of the individual epidemics during 2020–2022. PU is located in Mercer County, New Jersey, USA; the population comprises 5,267 undergraduate students, 2,946 graduate students, and around 7,000 faculty and staff members. For simplicity, we divided the epidemic into four time periods representing four semesters across two academic years: Fall 2020–2021 (2020 August 24–2021 January 1; Fig. 1A), Spring 2020–2021 (2021 January 16–2021 May 14; Fig. 1B), Fall 2021–2022 (2021 August 14–2021 December 31; Fig. 1C), and Spring 2021–2022 (2022 January 1–2022 March 18; Fig. 1D). Throughout the majority of the study period, all students, faculty and staff members who were physically present for more than 8 h on campus per week were required to participate in asymptomatic testing with varying frequencies. Asymptomatic individuals submitted self-collected saliva samples, from which the presence of SARS-CoV-2 was tested using reverse transcription polymerase chain reaction (PCR). Those who tested positive were required to isolate for at least 10 days after symptom onset or test date (whichever was longer) and were released when they had been at least 48 h with improving or resolving symptoms as per New Jersey Department of Health guidance; the isolation duration switched to 5 days on 2022 January 14, for all vaccinated people. PCR positives were exempt from asymptomatic testing for 90 days. Since 2022 March 7, asymptomatic testing frequencies decreased to once a month from once a week for individuals whose vaccine status is up-to-date. This in turn likely reduced the accuracy of surveillance; therefore, we chose to focus on epidemic patterns before this change was implemented. In Online Supplementary Fig. S1, we show the testing volumes over time—the testing volumes remained roughly constant within each semester, except when testing frequency changed, which we note in the paper. However, changes in testing frequency were not associated with sudden changes in cases, meaning that the patterns in case trajectories likely reflect patterns of spread, rather than testing behavior. Throughout the study period, contact tracing was also performed for positive cases to alert their close contacts to test more frequently and quarantine as applicable, according to the close contacts’ vaccination status, and to gather data that could help uncover clusters of transmission or superspreader events. Changes in testing frequency and other intervention measures throughout the study period reflected various factors, including the impact of COVID-19 cases on continuity of operations or continuity of teaching; on severity of disease on campus; the capacity of testing and the healthcare system; and hospitalization rates on campus and in the area. All data used in this analysis are publicly available on the PU COVID-19 Dashboard website: https://covid.princeton.edu/dashboard. Use of these data for this study was determined to be exempt from review by the Princeton Institutional Review Board.

## Descriptive analysis

During the *fall semester of the 2020–2021 academic year*, roughly 1,000 grad students and 2,000 faculty and staff members were present on campus and participated in asymptomatic testing. All classes were held virtually, and so only a few undergraduate students remained on campus (<300). Both undergraduate and graduate students were required to get tested twice a week, whereas faculty and staff members were required to get tested once a week. The number of cases remained relatively low throughout the semester with a peak occurring in early December, coinciding with the epidemic trajectory in Mercer County (Fig. 1A). A sudden decrease in the number of cases around Thanksgiving—a national holiday in the US during which many students travel off campus—partly reflects the reduced number of tests (3,852 and 2,972 asymptomatic tests performed on the week ending November 20 and 27, respectively). The highest number of cases was reported among faculty and staff members (=169), followed by graduate students (=41), and undergraduate students (=4). Even when we control for the differing population sizes among these groups, we find that a considerably larger amount of cases were reported among faculty and staff members (84.5 cases per 1,000) than from graduate students (41 cases per 1,000) (Online Supplementary Fig. S2A)—we exclude the undergraduate student population from this comparison due to a very low number of undergraduate students present on campus during this period.

In the beginning of the *spring semester of the 2020–2021 academic year*, the number of cases suddenly increased before classes started (Fig. 1B), reflecting ≈3000 undergraduate students returning to campus. Returning students were required to be tested upon arrival and quarantine for 7 days regardless of their returning location. Most classes remained virtual, and the testing protocol did not change (twice a week for undergraduate and graduate students, and once a week for faculty and staff members). Some smaller classes were held in-person, but required social distancing (thereby limiting the size of the class) and mask-wearing at all times. The number of cases persisted at similar levels to the fall semester and eventually decreased as classes ended and students went home—the decrease in the number of cases in PU also coincided with the decrease in the number of cases in Mercer County. The highest number of cases was reported among faculty and staff members (=111), followed by undergraduate students (=101), and graduate students (=29). This ordering is robust to differences in population sizes: 37 cases per 1000 among faculty and staff members, 34.3 cases per 1,000 among undergraduate students, and 14.5 cases per 1,000 among graduate students (Online Supplementary Fig. S2B).

For the *fall semester of the 2021–2022 academic year*, all students and faculty and staff members were required to be vaccinated with the COVID vaccine primary series, with very few medical and religious exemptions. By the beginning of the semesters, 97% of undergraduate students, 96% of graduate students, and 94% of faculty and staff members were vaccinated. Vaccinees were required to be tested once a week, while unvaccinated and partially vaccinated individuals were required to be tested twice a week. In-person classes and social events fully resumed on campus, though all individuals were required to wear masks indoors with a few exceptions (e.g. when eating or drinking, or when teaching any size of class if social distancing can be maintained at all times). The number of cases remained similar to previous semesters until November when cases began to increase, primarily among undergraduate students around Thanksgiving (Fig. 1C). In order to prevent transmission, testing frequency was increased to twice a week for undergraduate students on 2021 November 27; the size of nonacademic gatherings were also limited to 20 people. The number of cases decreased slightly as classes ended but soon increased again as the Omicron (BA.1) variant began to spread on campus and in Mercer County. The total number of reported cases per 1,000 remained high for all three population groups: 74 cases per 1,000 among faculty and staff members, 63.5 cases per 1,000 among graduate students, and 52.4 cases per 1,000 among undergraduate students (Online Supplementary Fig. S2C).

For the *spring semester of the 2021–2022 academic year*, all eligible students and faculty and staff members were required to have obtained booster vaccination. By the beginning of the semesters, 65% of undergraduate students, 71% of graduate students, and 82% of faculty and staff members were boosted. Undergraduate students were still required to be tested twice a week to prevent the additional spread of the Omicron variant. The number of cases remained high before classes began but decreased over time, following epidemic patterns in Mercer County (Fig. 1D). Coinciding with the decrease in the campus and local cases numbers, the gathering policy was updated on 2022 February 8 to allow food in events and event sizes were no longer limited to 20 people; in addition, the testing frequency was reduced to once a week (but not for unvaccinated individuals). Following the policy change, a large gathering event was held on campus, which resulted in an outbreak with high case numbers persisting until Spring Break (2022 March 5). The timing of this outbreak also coincided with a rapid increases in the Omicron subvariant BA.2 cases—the proportion of BA.2 subvariant reached 93.5% (372/398) compared to 26.9% (14/52) from the previous week. On 2022 March 7, mask mandates were lifted and testing frequency was reduced to once a month. Cases were largely concentrated among undergraduate students during this semester: 309 cases per 1,000 among undergraduate students, 106 cases per 1,000 among faculty and staff members, and 65.3 cases per 1,000 among graduate students.

### Comparisons of campus and community transmission

In order to compare the transmission dynamics in PU campus and in nearby communities, we calculate the Pearson correlation between the weekly numbers of cases from PU and those from nearby communities. Confidence intervals (CIs) are calculated using Fisher’s Z transformation, and the corresponding significance is calculated using asymptomatic *t* approximation (20). The standard method of calculating Perason correlations and the associated significance assumes that the samples are independent and identically distributed—these assumptions generally do not hold for epidemic time series data, which are autocorrelated and nonstationary. Nonetheless, the Pearson correlation has been the standard measure in epidemic time series analyses and can provide useful information about the strength of spatial coupling (21–24). We note correlation analyses do not provide direct evidence for causality and therefore should be interpreted with care—we later investigate the role of community transmission using a mathematical model.

Across the first three semesters, we find strong and significant correlations between the weekly numbers of cases from PU and those from Mercer County: fall 2020–2021 (ρ=0.79 [95% CI: 0.52–0.91; P<0.001], Fig. 1E); spring 2020–2021 (ρ=0.84 [95% CI: 0.60–0.94; P<0.001], Fig. 1F); and fall 2021–2022 (ρ=0.93 [95% CI: 0.84–0.97; P<0.001], Fig. 1G). These correlations are robust even when we stratify cases by the population, except for undergraduate students during Fall 2020, when most were not physically present on campus (Online Supplementary Fig. S3). However, the case patterns in PU were decoupled from those in Mercer County for the spring semester of the 2021–2022 academic year with unclear correlations (Fig. 1H): ρ=0.47 (95% CI: −0.18–0.83; P=0.15). Stratifying cases by subpopulation shows that case patterns in graduate students and faculty and staff members were still strongly correlated with case patterns in Mercer County (Online Supplementary Fig. S3). We also find strong correlations between the weekly logged numbers of cases from PU and those from other counties in New Jersey (Online Supplementary Fig. S4)—these correlations significantly decreased with distance from Mercer County in both spring 2020–2021 (ρ=−0.48 [95% CI: −0.75 to −0.06; P=0.03] for all cases and ρ=−0.51 [95% CI: −0.77 to −0.10; P=0.02] for faculty and staff cases) and fall 2021–2022 (ρ=−0.68 [95% CI: −0.86 to −0.35, P<0.001] for all cases and ρ=−0.74 [95% CI: −0.89 to −0.46, P<0.001] for faculty and staff cases). Across the first three semesters, both the total cases and faculty and staff cases showed similar levels of correlations with local cases. For the spring semester of the 2021–2022 academic year, we still find high correlations between faculty and staff cases and local cases throughout other counties (ρ>0.8 across all counties in New Jersey); however, the total cases exhibit considerably weaker correlations (Online Supplementary Fig. S4).

These correlations likely reflect commuting and contact patterns, and therefore we expect SARS-CoV-2 dynamics on campus to be correlated with those from nearby large cities as well. We find similarly strong correlations with New York City for the first three semesters: fall 2020–2021 (ρ=0.64 [95% CI: 0.26–0.85; P=0.003], Online Supplementary Fig. S5A); spring 2020–2021 (ρ=0.80 [95% CI: 0.53–0.93; P<0.001], Online Supplementary Fig. S5B); fall 2021–2022 (ρ=0.89 [95% CI: 0.73–0.96; P<0.001], Online Supplementary Fig. S5C); and spring 2021–2022 [ρ=0.50 (95% CI: −0.14 to −0.85; P=0.1], Online Supplementary Fig. S5D).

A similar picture emerges for Philadelphia except for spring 2020: fall 2020–2021 (ρ=0.87 [95% CI: 0.68–0.95; P<0.001], Online Supplementary Fig. S6A); spring 2020–2021 (ρ=0.27 [95% CI: −0.24–0.66; P=0.30], Online Supplementary Fig. S6B); fall 2021–2022 (ρ=0.89 [95% CI: 0.74–0.96; P<0.001], Online Supplementary Fig. S6C); and spring 2021–2022 (ρ=0.46 [95% CI: −0.19 to −0.83; P=0.15], Online Supplementary Fig. S6D). Including counties from New York and Pennsylvania states into the spatial correlation analysis yields additional insights (Online Supplementary Fig. S7): epidemic dynamics were highly synchronized across all counties in fall 2020–2021 and became less synchronized over time. These correlations significantly decreased with distance in spring 2020–2021 (ρ=−0.36 [95% CI: −0.49 to −0.21; P<0.001]) and fall 2021–2022 (ρ=−0.58 [95% CI: −0.68 to −0.46; P<0.001]). These variations likely reflect differences in vaccination levels and the timing of the introduction of the Omicron variant.

Finally, mass testing allows us to infer the ratio between the weekly numbers of cases per 1,000 from Princeton and those from Mercer County—we expect this ratio to remain constant around 1 over time when (1) there is random, homogeneous mixing between the campus and community and (2) testing patterns remains constant in both places within each semester. In this case, the majority of infections in PU campus would be caused by community transmission owing to its small population size. Instead, we find that the ratio generally hovers above 1 during the fall semester of the 2020–2021 academic year even though there was little-to-no documented transmission on campus (Fig. 1I). This pattern likely reflects a higher testing rate on campus, thereby resulting in a higher case ascertainment rate. For the most part of the spring semester of the 2020–2021 academic year and the fall semester of the 2021–2022 academic year, the case ratios hover around 1 (Fig. 1J and K). Deviations from the one-to-one ratio were often associated with large campus events, such as school holidays and the beginning and end of semesters. An increase in this ratio at the end of November 2021 was associated with the campus outbreak before Thanksgiving followed by an introduction of the Omicron variant in December—this deviation indicates an increase in the amount of transmission on campus. During the spring semester of the 2021–2022 academic year, the ratio between PU cases and Mercer County cases increased above one due to a large outbreak on campus (Fig. 1L); notably, we did not see an increase in Mercer County cases (Fig. 1D), meaning that there was little-to-no transmission from campus to local community.

## Mathematical modeling of past outbreaks

We use a discrete-time, individual-based model to simulate the spread of SARS-CoV-2 on the PU campus. This model was initially developed and used throughout the pandemic to inform policy decisions in PU, including the frequency of asymptomatic tests and the number of isolation beds required. We continuously updated the model to reflect changes in school settings (e.g. students returning back to campus after a virtual semester) as well as intervention measures (e.g. vaccination in fall 2021 and booster shots with the emergence of the Omicron variant). Here, we present a generic and parsimonious version that encompasses sufficient details to characterize the overall spread of SARS-CoV-2 in PU without an over-proliferation of parameters. The model consists of four main components simulated on a daily time scale: (1) infection and transmission dynamics, (2) sampling and testing protocols, (3) isolation protocols, and (4) vaccination dynamics, including waning immunity and booster shots. Previous versions of the model included contact tracing, but we exclude it in this model for simplicity.

Infection processes are modeled based on standard compartmental structures (Online Supplementary Fig. S8). Once infected, susceptible individuals remain in the exposed stage for $D_{e}=2$ days on average, during which they cannot transmit or test positive. Exposed individuals then enter the presymptomatic stage, during which they can test positive and transmit infections for $D_{p}=3$ days on average. Presymptomatic individuals can then either remain asymptomatic with probability $p_{a}=0.4$ or develop symptoms with the remaining probability of $1-p_{a}=0.6$; both asymptomatic and symptomatic individuals are assumed to have the same duration of infectiousness ($D_{s}=3$) and equal transmission rates. Recovered individuals are assumed to be immune to reinfections throughout a semester. Presymptomatic, symptomatic, and asymptomatic infection stages are further divided into two subcompartments. Dividing each infection stage into subcompartments allows for the duration of infection to have narrower (and more realistic) distributions than the exponential distribution (25). Transitions between each (sub)compartments are modeled using a Bernoulli process with probabilities that match the assumed means (26): more specifically, transition probabilities are equal to $1-\exp\;(-\delta_{x})$, where $\delta_{x}=-\log\;(1-n/D_{x})$ represent the transition rate from stage *X* and *n* represents the number of subcompartments. Assumed parameters are broadly consistent with other models of SARS-CoV-2 (25, 27).

Transmission processes are modeled by first setting the contact reproduction number $R_{\mathrm{contact}}$, which we define as the average number of infectious contacts an infected individual would make throughout the course of their infection; here, infectious contacts refer to contacts that would result in infection when the contacted individual is susceptible to infection. We note that the definition of the contact reproduction number $R_{\mathrm{contact}}$ is similar to the standard definition of basic reproduction number $R_{0}$. The main difference is that the contact reproduction number models the number of total contacts, rather than infections. Since infected individuals make their contacts at random with replacement, the same susceptible person could be contacted multiple times by the same or different infected individual during a time step—all these overlapping contacts will result in one infection. Therefore, the number of actual infections may be smaller than the number of contacts, especially since contacts can also land on nonsusceptible individuals. We also note that the contact reproduction number implicitly accounts for all intervention measures that we do not model explicitly, such as social distancing and contact tracing—therefore, $R_{\mathrm{contact}}$ is similar to the effective reproduction number, which typically accounts for intervention efforts. However, our contact reproduction number does not account for the effects of asymptomatic testing or vaccination, which are modeled separately. We further decompose $R_{\mathrm{contact}}$ into presymptomatic $R_{p}=\beta_{p}D_{p}$ and (a)symptomatic $R_{s}=\beta_{s}D_{s}$ reproduction numbers, where $\beta_{p}$ and $\beta_{s}$ represent the corresponding infectious contact rates during presymptomatic and (a)symptomatic stages, respectively. Presymptomatic and (a)symptomatic reproduction numbers are calculated based on the assumed value of the proportion of presymptomatic transmission $p_{p}=0.5$: $R_{p}/R_{s}=p_{p}/(1-p_{p})$. On each day, all infected individuals who have not yet been isolated then make infectious contacts at random to anyone on campus; the number of infectious contacts are drawn from a negative binomial distribution with a mean of either $\beta_{p}$ or $\beta_{s}$ and an overdispersion parameter of k=0.1 to account for the possibility of superspreading events (28).

In reality, the contact structure among the campus population is likely more structured, exhibiting strong assortativity. For example, undergraduate students are more likely to mix with other undergraduate students, rather than graduate students or faculty and staff members. Even among undergraduate students, students are more likely to mix with their close friend group than with other students. On one hand, assortative mixing may lead to faster epidemic growth within certain population groups. On the other hand, it can also make the disease more difficult to spread among other groups that have lower contact rates. Therefore, predicting the impact of structured contact network requires more detailed information about whether the majority of cases were infected at random or from certain groups of the campus population. For simplicity, we assume random mixing throughout the paper—nonetheless, allowing for overdispersion in transmission is expected to emulate variability in epidemic growth rates driven by complex contact structures (29).

We also rely on cases from Mercer County to crudely capture community dynamics. In particular, we assume that infectious contacts from local or regional community can be made at random to anyone on campus. These contacts are modeled using a Poisson distribution with a time-varying mean, which is calculated by multiplying the daily number of cases by the community contact rate θ and the population size on campus *N*. More precisely, θ is the probability that an infected individual from the community makes an infectious contact with an individual on campus per capita campus population. By further multiplying this probability with the population size *N*, we are essentially assuming a density dependent contact, where a higher population size on campus leads to more infections from the community. We further shift community contacts by 1 week to account for reporting delays. Infectious contacts, whether made by individuals on campus or from outside, result in infection only when the contacted individuals are susceptible; when the contacted individuals are vaccinated, and therefore partially susceptible to infection, they have a reduced probability of infection corresponding to their susceptibility (discussed later).

All individuals on campus are assumed to follow a predetermined asymptomatic testing plan at a fixed frequency—for example, under weekly testing, one individual can get sampled on days 1, 8, 15, and so forth, while another individual get sampled on days 2, 9, 16, and so forth. We assume that test results come back after one day. Symptomatic individuals can choose to take rapid PCR tests (with results returning on the same day) with a given probability on each day until their symptoms resolve—this probability is set to 1 for simulations presented in the main text. We further assume that symptomatic individuals are isolated immediately when they submit their samples until they receive negative results. All individuals who test positive are required to isolate (following the same isolation rule as described earlier) and are exempt from asymptomatic testing for 90 days—while this assumption reflects the isolation policy in PU during the investigation period, it may be inapplicable in studying institutional outbreaks in general. Isolated individuals are assumed to no longer transmit infections. We assume that PCR tests can detect infections from individuals who are in presymptomatic, symptomatic, and asymptomatic stages with 95% sensitivity and 100% specificity.

The 95% sensitivity assumption may seem too high. For example, Hellewell et al. (30) estimated that the probability of detecting an infection from a PCR peaks at 77% (54–88%) 4 days after infection, and decreases to 50% (38–65%) by 10 days after infection. These estimates are considerably lower than our assumption because their estimates implicitly account for the latent period. At the individual level, we assume that an infected individual has no detectable infection (0% sensitivity) during their latent period and 95% sensitivity during their various stages of infectious periods. At the population level, this assumption translates to a peak sensitivity of 92% by 4 days after infection, decreasing to 20% sensitivity by 10 days after infection. Our assumption leads to a much lower PCR sensitivity 10 days after infection because we only model the PCR sensitivity during the infectious period. In reality, PCR can also detect viral nucleic acids even after a person stops shedding infectious virus, but since these nucleic acids cannot contribute to person-to-person transmission or affect the effectiveness of the isolation strategy, we did not include this component in our model.

As most students, as well as faculty and staff members, had received two doses of vaccination in the beginning of fall 2021, we do not distinguish the first and second doses. Instead, we assume that all vaccinated individuals have 90% reduced susceptibility and 20% reduced transmissibility at the beginning of the semester—these assumptions are consistent with recent estimates by Prunas et al. (31) that vaccination with BNT162b2 reduces susceptibility by 89.4% (95% CI: 88.7–90.0%) and infectiousness by 23.0% (95% CI: −11.3 to −46.7%) against the Delta variant. Based on Tartof et al. (32), vaccine efficacy against susceptibility is allowed to exponentially wane from 90 to 50% in 20 weeks (and continues to wane at the same rate) for each vaccinated individual; vaccine efficacy against transmissibility is also allowed to wane at the same rate (i.e. from 20 to 11% in 20 weeks). We note that these assumptions are specific to the Delta variant—we discuss vaccine effectiveness against the Omicron variant later on.

In this study, we use this model to retrospectively analyze past outbreaks. First, we try to match our model to epidemic patterns seen on campus for the first three semesters, during which there was limited campus transmission, by varying the contact reproduction number $R_{\mathrm{contact}}$ and the amount of community contact θ and holding all other parameters constant. For each parameter combination, we simulate 100 epidemic trajectories and calculate the sum of squared differences between the weekly numbers of the observed and predicted positive cases. The population size and testing frequencies (with twice weekly testing modeled as testing every 3 days) are set to reflect realistic campus settings. Although we account for heterogeneity in the number of individuals in each population group on campus (i.e. undergraduate students, graduate students, and faculty and staff members) and their respective testing patterns (e.g. twice a week for undergraduate and graduate students and once a week for faculty and staff members during fall and spring, 2020), we assume, for simplicity, that all other parameters are equal across different groups. We further assume that the population mixes homogeneously. While these assumptions are most parsimonious, epidemiological parameters and mixing patterns likely differ across groups (e.g. undergraduate students are more likely to infect undergraduate students and also remain asymptomatic). Therefore, our model parameters describe average dynamics across different groups and must be interpreted with care.

**Fig. 2.**
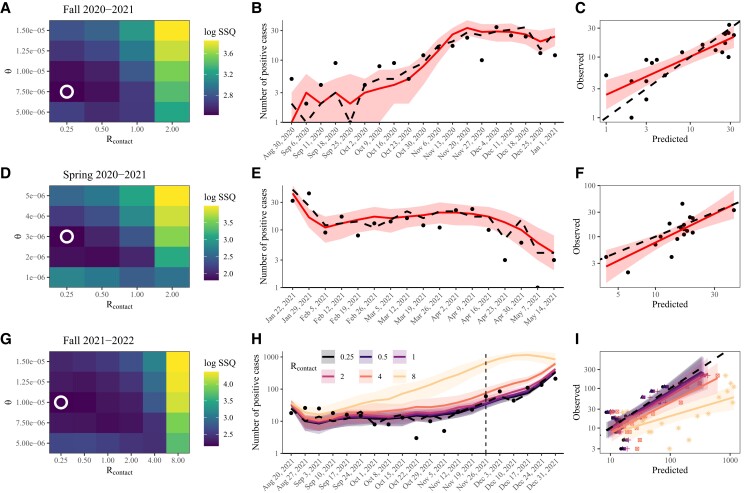
Retrospective analysis of past SARS-CoV-2 outbreaks in PU campus. A, D, G) Time series comparisons of model predictions with observed data across ranges of contact reproduction number $R_{\mathrm{contact}}$ and scaling parameter for community contact θ. For each parameter combination, we simulate the model 100 times and calculate the sum of squared differences (SSQ) between the reported number of positive cases and the model-predicted number of positive cases. Heat maps represent medians of the logged sum of squared differences. Circles represent the best fitting parameter set. B, E, H) Model predictions. Solid lines represent median predictions. Dashed lines represent a realization with the least sum of squared errors. Shaded areas represent 90% quantiles for the best matching parameter set. Points represent the observed data. C, F, I) Correlations between model predictions with observed data. Colored solid lines and shaded areas represent the estimated linear regression lines and the associated 95% CIs. Dashed lines represent the one-to-one line.

For *fall 2020–2021*, we simulate the model assuming 3,000 individuals (1,000 graduate students and 2,000 faculty and staff members) on campus with 1,000 of them participating in asymptomatic testing twice a week. We find that a low level of contacts $R_{\mathrm{contact}}=0.25$ and a small amount of community contact $\theta =7.5\times 10^{-6}$ is most consistent with the observed epidemic dynamics in fall 2020 (Fig. 2A). With these parameters, the model is able to capture the rise and fall in the number of cases with the exception of a sudden decrease in the number of cases around Thanksgiving, which we do not model explicitly (Fig. 2B). The median predictions are positively correlated with the observed dynamics (ρ=0.83; 95% CI: 0.61–0.93; Fig. 2C). Although a wide range of assumptions about the levels of community contact θ are consistent with the observed dynamics, our simulations preclude high levels of contact, $R_{\mathrm{contact}}>2$ (Online Supplementary Fig. S9). Distancing measures on campus and contact tracing efforts likely contributed to lowering contact levels $R_{\mathrm{contact}}$.

For *spring 2020–2021*, we simulate the model assuming 8,000 individuals (3,000 undergraduate students, 2,000 graduate students, and 3,000 faculty and staff members) on campus with 5,000 of them participating in asymptomatic testing twice a week. We further assume that 4,000 individuals (3,000 undergraduate students and 1,000 graduate students) returned to campus over 14 days (2021 January 16–2021 January 29). In the beginning of the semester, all returning students were required to quarantine in their rooms for 14 days and tested upon returning by the university—in our model, this was implemented by preventing returning students from getting infected or infecting other individuals. Finally, to match the initial influx of cases, we assume that 1% of both returning and on-campus populations are infected at the beginning of simulation (2021 January 16).

A similar set of parameters can capture the observed dynamics in spring 2020–2021. The best matching parameter predicts considerably lower levels of community contact $\theta =3\times 10^{-6}$ (Fig. 2D), but a wide range of parameters are consistent with the observed dynamics as before (Supplementary Fig. S10). Simulations also preclude high $R_{\mathrm{contact}}>2$ again, suggesting that transmission between students were likely limited even though they had returned to campus—the absence of in-person teaching is likely to have contributed to lowering $R_{\mathrm{contact}}$. We also find that initial infections (e.g. from returning students) are required to match relatively high levels of cases in the beginning of semester (Fig. 2E). Once again, the predicted and the observed numbers of cases are positively correlated (ρ=0.62; 95% CI: 0.20–0.85; Fig. 2F).

For *fall 2021–2022*, we assume 13,000 individuals are present on campus (5,000 undergraduate students, 2,000 graduate students, and 6,000 staff and faculty members) with 98% of them vaccinated—here, vaccine-derived immunity is allowed to wane over time to ask whether the increase in the number of cases around November is consistent with the dynamics predicted by immunity waning. Vaccinated individuals are tested every week, whereas unvaccinated individuals are tested every 3 days. We further assume 5,000 undergraduate students returned to campus over 16 days (2021 August 14–2021 August 29). All students were required to test upon return and quarantine until they received a negative test result; for simplicity, we only model the testing process in our simulation (without quarantine) given a short testing delay. Finally, we assume that 0.5% of both returning and on-campus populations are infected at the beginning of simulation (2021 August 14). We limit our model comparison to November 26 before the Omicron variant was introduced on campus.

The per capita numbers of cases during fall 2021 (before a large outbreak) were considerably lower than to those during previous semesters. Nonetheless, we find that higher levels of community contact $\theta =1\times 10^{-5}$ are required to explain the observed dynamics due to a decreased susceptibility from vaccination (Fig. 2G). We note that the parameter θ necessarily depends on our assumed vaccine efficacy against susceptibility, and θ would decrease if we assume a lower vaccine efficacy. Nonetheless, the amount of community contact would still need to be higher than previous semesters as long as the vaccine provides some protection against infection and onward transmission.

While $\theta =1\times 10^{-5}$ and $R_{\mathrm{contact}}=0.25$ gives the best matching parameter set with a median logged sum of squared errors of 8.61 (95% quantile: 5.80–11.6), other parameter sets also give nearly identical fits (Fig. 2H; Online Supplementary Fig. S11). Comparing simulations across a wide range of $R_{\mathrm{contact}}$ (0.25–8) with $\theta =1\times 10^{-5}$ further illustrates that the predicted dynamics are largely insensitive to $R_{\mathrm{contact}}$ until November 26 (Fig. 2H). All simulations shown in Fig. 2H, except for the $R_{\mathrm{contact}}=8$ scenario, are similarly correlated with the observed numbers of cases (Fig. 2G). While the logged sum of squared errors increases with $R_{\mathrm{contact}}$ (Fig. 2G), these patterns are likely driven by the discrepancy around fall break (week ending October 26) when the number of cases decreased suddenly, rather than a lack of fit—we did not explicitly model holiday effects for simplicity. Extremely high vaccination rates and frequent testing likely limited transmission on campus, making epidemic dynamics largely insensitive to $R_{\mathrm{contact}}$ even at a reasonably high value of $R_{\mathrm{contact}}=4$.

These simulations suggest that an increase in the number of cases in November can be explained by a combination of waning immunity alone without requiring additional changes in transmission dynamics (note we do not allow θ or $R_{\mathrm{contact}}$ to vary over time)—we see that extending the simulation beyond November 26 still captures the increase in cases. When we exclude immune waning from the model, predicted epidemic dynamics exhibit slower growth and require even higher values of campus and community contact rates ($R_{\mathrm{contact}}$ and θ) to qualitatively match the observed dynamics (Online Supplementary Fig. S12)—even so, the logged sum of squared differences are generally higher (with median logged sum of squared differences ranging from 7.2 to 33.9 for the same parameter regime). Thus, combining some amount of immune waning and high campus and community contact rates likely best explains the epidemic growth near the end of the semester. We note that other factors, such as changes in behavior, could have also contributed to the increase in the numbers of cases.

Projecting the model beyond November 26 implies that we would have seen a similar growth in the number of cases if conditions remained constant even without the introduction of the Omicron variant. In other words, the Delta strain would have continued to spread on campus at a similar rate if the semester were to (hypothetically) continue until January without additional interventions due to immune waning and growing cases in the community (Fig. 2H). In reality, the situation was more complex: testing frequencies increased and social gatherings were limited in response to an increase in the number of cases. These interventions—as well as students returning back home as classes ended—likely would have reduced contact rates (and therefore transmission of the Delta variant). This reduction in transmission was likely counterbalanced by the introduction of the Omicron variant and its high transmissibility and immune evasion, leading to similar and persistent growth in the number of cases.

## The spread of the Omicron variant on campus

Epidemiological conditions and intervention measures changed throughout the spring semester of the 2021–2022 academic year. We therefore extend the model to account for these alterations and focus on the outbreak patterns among undergraduate students. First, based on Ferguson et al. (33), we assume that two and three doses of vaccines reduce susceptibility against the early Omicron variant by 10 and 70%, respectively. We also assume that the transmissibility of Omicron is reduced proportionally following the previously assumed 90:20 ratio for the Delta variant; in other words, two and three doses of vaccines reduce transmissibility by 2.2 and 15.6%, respectively. The immunity from the third dose is assumed to take 7 days to develop in our model (34) and wane at the same rate as before (in this case, 70 to 39% in 20 weeks). Finally, the isolation period was reduced to 5 days—the actual change was implemented on 2022 January 14 but we keep the 5-day isolation period throughout our simulations, which begins on 2022 January 7, for simplicity.

Here, we use the extended model to try to understand the drivers of a large campus outbreak that happened on the week ending 2022 February 18 (Fig. 1D). First, we ask whether changes in testing frequency from biweekly to weekly and an increased reproduction number can explain the outbreak. The increase in the reproduction number can reflect increased contact rates following changes in distancing policy as well as increased transmissibility of the BA.2 subvariant—we do not explicitly distinguish the cause of the increase in the reproduction number. We do so by simulating the model forward across a range of contact reproduction numbers that are consistent with previous estimates ($R_{\mathrm{contact}}=2-6$) and introducing a 20–100% increase in the contact reproduction number on 2022 February 8, with changes in the testing frequency. To match the realistic campus setting, we assume that 700 students are present on campus as of 2022 January 1, and the remaining 4,300 students come back to campus across 28 days. Based on known vaccination statuses, we assume that 99% of students are vaccinated with 60% of them being boosted as of 2022 January 1. Since all students were required to receive booster shots before returning to campus, we assumed that 70 booster shots were given on each day—this assumption allows all students to be boosted in 28 days. To match the high numbers of cases on the week ending 2022 January 7, we assume 14% of the students present on campus are infected as of 2022 January 7 (roughly 100/700). To account for students who were infected with the Omicron variant during the fall semester, we assume that 100 students are already immune to Omicron infection at the beginning of the spring semester—this roughly corresponds to the number of PU cases that were reported in December.

**Fig. 3.**
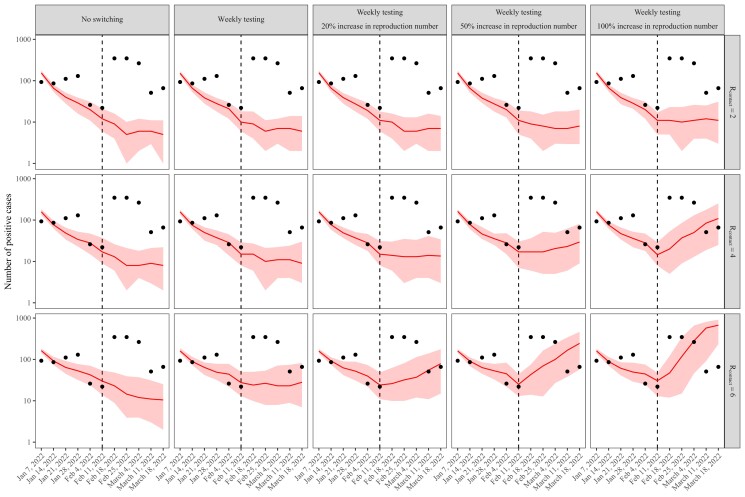
The impact of changes in testing frequency and an increased reproduction number on the spread of the Omicron variant. Solid lines represent median predictions. Shaded areas represent 90% quantiles across 100 simulations. Points represent the observed data. Vertical dashed lines represent the week including 2022 February 8, when distancing and testing policies were updated in PU campus. For each row, we assume a fixed value of baseline contact reproduction number $R_{\mathrm{contact}}$ ranging from 2 to 6 across rows. Then, we simulate increase in $R_{\mathrm{contact}}$ at the time of policy change (indicated by column labels).

In the absence of changes in testing frequency or an increased reproduction number, the model predicts the number of cases among undergraduate students to continue to decrease over time (Fig. 3). Changes in testing frequency alone have negligible impact on the overall dynamics; when the baseline contact reproduction number $R_{\mathrm{contact}}$ is sufficiently high ($R_{\mathrm{contact}}=6$), changing testing frequency from biweekly to weekly causes the weekly case numbers to stay at a constant level (instead of decreasing). Additional increases in the reproduction number (alongside the changes in testing frequency) can cause the case numbers to further increase, but we are unable to match the observed dynamics even with a 100% increase in the reproduction number. Indeed, a >10-fold increase in the numbers of cases between the weeks ending 2022 February 11 and 18, would require an unrealistically high increase in the contact reproduction number to explain. These simulations indicate that changes in distancing and testing policies and the increased transmissibility of the BA.2 subvariant alone are unlikely to be the direct causes of the outbreak.

We considered the possibility that the Omicron variant can have shorter latent and infectious periods by decreasing the mean duration of latent, presymptomatic, and (a)symptomatic stages of infection by 0.5 days (therefore a total of 1.5 reduction in the duration of infection). In this case, a shorter generation interval can lead to faster growth rate given the same values of $R_{\mathrm{contact}}$ (35). However, we find that the effects of shorter infection has small effects on the overall dynamics (Online Supplementary Fig. S13).

We also considered the possibility that the vaccine effectiveness against the Omicron variant might be lower by repeating the same analysis with 30% effectiveness against infection (36). When the baseline $R_{\mathrm{contact}}$ is low ($R_{\mathrm{contact}}=2$), increasing $R_{\mathrm{contact}}$ still does not increase the number of cases sufficiently. When we assume an intermediate value of $R_{\mathrm{contact}}=4$, the model does a better job at capturing the dynamics but it does so by overestimating the trough before the policy change and underestimating the peak after the policy change. When we assume a high value of $R_{\mathrm{contact}}=6$, the model overestimates both the trough and the peak (Online Supplementary Fig. S14).

**Fig. 4.**
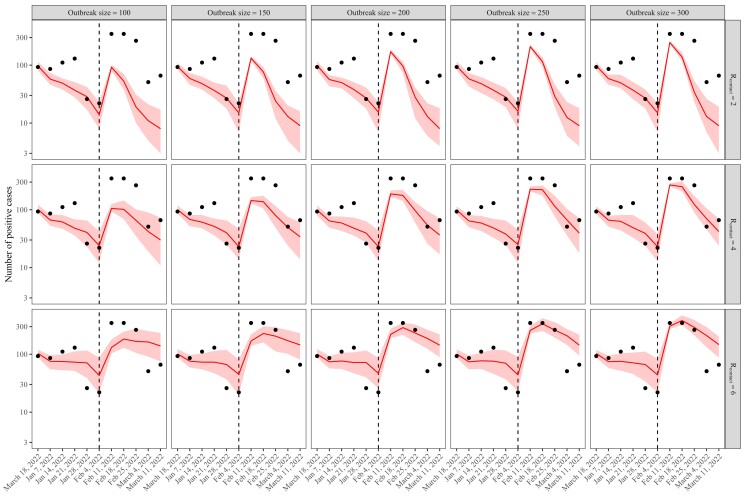
The impact of large superspreading events on the spread of the Omicron variant. Solid lines represent median predictions. Shaded areas represent 90% quantiles across 100 simulations. Points represent the observed data. Vertical dashed lines represent the week including 2022 February 8, when distancing and testing policies were updated in PU campus.

Instead, we consider the role of superspreading events in driving a large Omicron outbreak by simulating 100–300 infections happening on the same day (2022 February 12, the weekend following the policy change). We still include changes in testing to reflect realistic settings on campus but do not model the increase in the reproduction number to test the sole effects of superspreading events. In contrast to previous simulations (Fig. 3), which showed persistent growth in cases following the increase in the reproduction number, an epidemic driven by a superspreading event plateaus and decays quickly (Fig. 4). In this case, moderate values of baseline reproduction numbers permit a small amount of onward transmission, which can sustain the epidemic for a few weeks, but the reproduction number is not high enough to cause the epidemic to keep growing. Overall, the observed patterns in cases are more consistent with the epidemic dynamics driven by superspreading events. This is also consistent with the observation that this outbreak was associated with a large gathering event on campus.

## Discussion

Here, we analyze SARS-CoV-2 outbreaks on the PU campus between fall 2020 and early 2022. We demonstrate strong spatiotemporal correlations between the patterns of spread of SARS-CoV-2 on campus and those from surrounding communities. These correlations decreased with distance from Mercer County in fall 2021–2022, indicating weaker spatial coupling in the dynamics. Mathematical modeling further suggests limited transmission between the university population during fall and spring semesters of the 2020–2021 academic year and an increased frequency of infective community contacts during the fall semester of the 2021–2022 academic year, compared to previous semesters. An increase in the number of cases by the end of November 2021 is consistent with the increase in the levels of community cases and waning immunity. Finally, our analysis highlights the potential role of superspreading events in driving the spread of the Omicron variant on the PU campus.

Although previous outbreak reports from other universities primarily focused on within-campus transmission (37, 38), a few studies identified off-campus infections as an important source of transmission (39, 40). For example, extensive modeling efforts from Cornell University demonstrated an increase in the amount of transmission from outside the university campus during fall 2021 and found that community transmissions are the biggest risk for faculty and staff members (14). Our study further extends these findings in demonstrating a strong spatiotemporal correlation in the spread of SARS-CoV-2 between university campuses and surrounding communities; however, when campus transmission is sustained, community coupling becomes less important. The degree to which community coupling affects campus transmission also depends on the campus. Although PU is located in a small county (Mercer County) with a population of 390,000 (www.census.gov), it is located near large cities, such as New York City and Philadelphia, which can drive infections in smaller cities nearby (41). For example, contact tracing efforts from Boston University, which is located in a large metropolitan area, found that more than 50% of infections among Boston University affiliates with known exposures could be attributed to sources outside of the university campus (40). In contrast, other university campuses that are far from urban areas may experience weaker community coupling. The degree of coupling will also depend on intervention measures in surrounding communities and on campus. Understanding these heterogeneities is critical for preventing future campus outbreaks.

Our analysis also suggests that comparing the ratios between the cases on university campuses and neighboring communities can also provide a useful measure for how well a university campus is controlling the epidemic; however, that this ratio needs to be interpreted with caution, as it is sensitive to changes in testing patterns as well as the numbers of students on campus. For example, the ratios of cases can suddenly change during holidays when students are away from campus. Future studies could combine viral phylogenetic data to better understand spatial patterns of SARS-CoV-2 on campus.

There are several limitations to our analysis. While we demonstrate strong spatiotemporal correlation in the spread of SARS-CoV-2, correlations do not provide causal link for community transmission. For example, even if there was no mixing between campus and community populations, two populations can exhibit correlated epidemic dynamics if both populations are changing behavior in similar ways, reflecting changes in intervention measures in the greater New Jersey area. However, it seems biologically implausible that there would be no mixing between the two populatins—other studies have also identified community transmission to be an important source of SARS-CoV-2 infections on campus (39, 40). Our mathematical modeling illustrates that community transmission can explain the observed dynamics in PU campus, but we are not able to infer the direction of causality—that is, our analysis does not rule out the possibility that transmission on campus drove infections in nearby communities (as opposed to community transmission driving on-campus infections). However, seeding from campus is unlikely: intervention measures on campus (e.g. frequent asymptomatic testing, contact tracing, and virtual classes during fall and spring semesters of 2020) likely limited onward transmission on campus. In addition, even during periods of large Omicron outbreaks on campus in early 2022, the number of COVID-19 cases in Mercer County remained low, implying limited transmission from campus to community. Decreasing patterns in epidemic correlations with distance further highlight the role of spatial spread in driving dynamics of SARS-CoV-2—such patterns are consistent with spatial spread of many other respiratory pathogens (21, 22, 24).

Our mathematical model relies on simplifying assumptions. For example, we assume conservatively that the entire university populations mix homogeneously and have identical campus and community contact rates (captured by $R_{\mathrm{contact}}$ and θ, respectively). This assumption can lead to the fastest epidemic growth rates because transmission is not limited by the size of the contact network—in other words, our estimates of the reproduction will be necessarily low, making the epidemic easier to control. In reality, increases in cases were often associated with specific transmission clusters, suggesting heterogeneity in transmission patterns. Contact levels also likely differ between different groups: for example, faculty and staff members are more likely to interact with community members than undergraduate students and would be at a higher risk for community infections (14). We also do not account for explicit changes in behavior on campus and assume constant $R_{\mathrm{contact}}$ throughout each semester. Instead, we implicitly account for behavioral changes in the community by modeling community transmission to campus as a function of community case numbers. While we cannot rule out the possibility that behavioral changes on campus could have contributed to various epidemics (e.g. the Omicron wave beginning in the fall semester of the 2021–2022 academic year), we were able to capture the majority of epidemic patterns without modeling them—when the majority of transmission is caused by imported cases from the community, we expect behavioral changes on campus to have relatively weaker effects on overall transmission dynamics. We also do not explore parameter uncertainty, which can lead to underestimation of overall uncertainty (42). We also note that intervention measures that were introduced to PU may not necessarily be applicable in other institutions.

Despite the simplicity of the analysis, our study provides important lessons for controlling SARS-CoV-2 and similar outbreaks on university campuses in general. First, our analysis highlights the power of mass asymptomatic testing for epidemic measurement and planning—even if PCR testing may have lower sensitivity than what we assumed here (30), mass asymptomatic testing can still help track ongoing epidemic dynamics in real time. Combining other intervention measures, such as social distancing, mask-wearing, and vaccination, can help provide some measures to consider for restoring operations on campuses—but the extent to which these interventions are implemented will necessarily depend on resource availability. Second, we expect immunity waning and superspreading to continue to play important roles in driving campus transmission—keeping vaccine statuses up-to-date within the campus community will be critical moving forward. Third, the safe reopening of a university campus must consider the spread of SARS-CoV-2 within the surrounding community as they can both potentially drive transmission in each other—however, the degree to which infections spread from campus to community remains uncertain. Finally, intervention measures placed on campuses must continue to adapt and change to reflect changes in epidemiological conditions. We note that the generality of our conclusions will necessarily depend on specific campus settings.

The emergence of new variants—in particular, their ability to evade prior immunity and transmit better—continues to add uncertainty to the future controllability of the ongoing SARS-CoV-2 pandemic. Nonetheless, as population-level immunity increases (either due to infection or vaccination), we are transitioning to an endemic phase, during which COVID-19 is expected to become less severe (11). Understanding the the landscape of SARS-CoV-2 immunity and its impact on evolutionary dynamics will be critical to predicting future outbreak dynamics (1, 13).

## Supplementary Material

pgad201_Supplementary_Data

## Data Availability

All data and code are stored in a publicly available GitHub repository (https://github.com/parksw3/university-covid).
