# Correction to: Relative role of community transmission and campus contagion in driving the spread of SARS-CoV-2: Lessons from Princeton University

**DOI:** 10.1093/pnasnexus/pgae180

**Published:** 2024-05-03

This is a correction to: Sang Woo Park, Irini Daskalaki, Robin M Izzo, Irina Aranovich, Aartjan J W te Velthuis, Daniel A Notterman, C Jessica E Metcalf, Bryan T Grenfell, Relative role of community transmission and campus contagion in driving the spread of SARS-CoV-2: Lessons from Princeton University, *PNAS Nexus*, Volume 2, Issue 7, July 2023, pgad201, https://doi.org/10.1093/pnasnexus/pgad201

After publication it was discovered that this paper was missing a statement acknowledging compliance with the *PNAS Nexus* Human and Animal Participants and Clinical Trials policy:

Use of these data for this study was determined to be exempt from review by the Princeton Institutional Review Board.

This error has been corrected in the original article.

