# Supplementary material for: Relative role of community transmission and campus contagion in driving the spread of SARS-CoV-2: Lessons from Princeton University
Source: PNAS Nexus. 2023 Jul 3;2(7):pgad201. doi: 10.1093/pnasnexus/pgad201 (PMC10338902; doi:10.1093/pnasnexus/pgad201)
Supplement: pgad201_Supplementary_Data [file pgad201_supplementary_data.pdf]

Supplementary Materials

Relative role of community transmission and campus contagion in driving the spread of SARS-CoV-2: lessons from Princeton University

Sang Woo Park[1] Irini Daskalaki[2] Robin M. Izzo[3] Irina Aranovich[4] Aartjan J.W. te Velthuis[5] Daniel A. Notterman[5] C. Jessica E. Metcalf[1,6] Bryan T. Grenfell[1,6]

1 Department of Ecology and Evolutionary Biology, Princeton University, Princeton, NJ, USA

2 University Health Services, Princeton University, Princeton, NJ, USA

3 Environmental Health and Safety, Princeton University, Princeton, NJ, USA

4 Princeton University Clinical Laboratory, Princeton University, Princeton, NJ, USA

5 Department of Molecular Biology, Princeton University, Princeton, NJ, USA

6 Princeton School of Public and International Affairs, Princeton University, Princeton, NJ, USA

Corresponding author: swp2@princeton.edu

Supplementary Figures

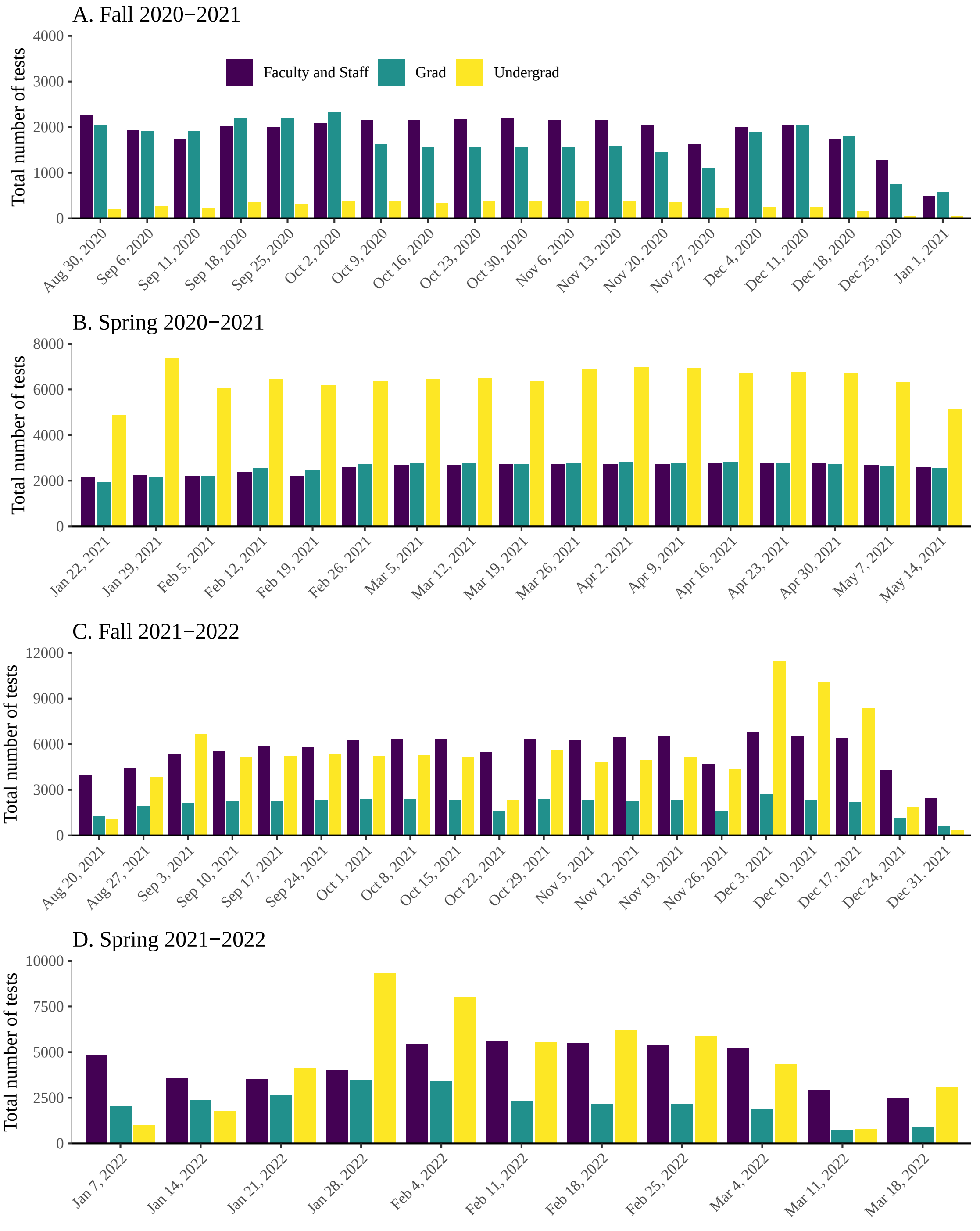

Figure S1: **The weekly number of PCR tests stratified by subpopulations.**

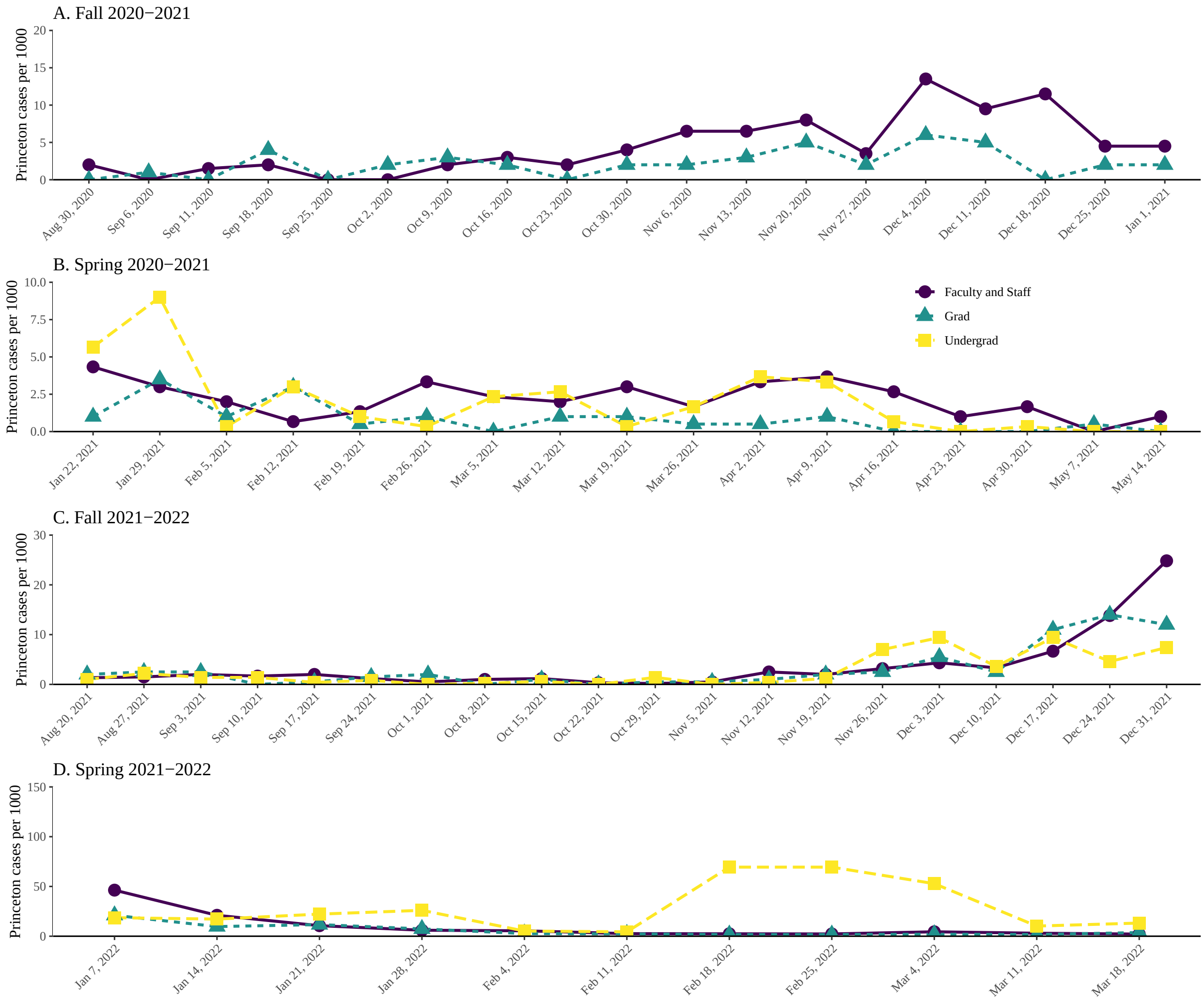

Figure S2: **The weekly number of cases per 1000 stratified by subpopulations.** The case trajectory for undergraduate students was omitted for the fall semester of 2020–2021 academic year due to a very low number of undergraduate students present on campus.

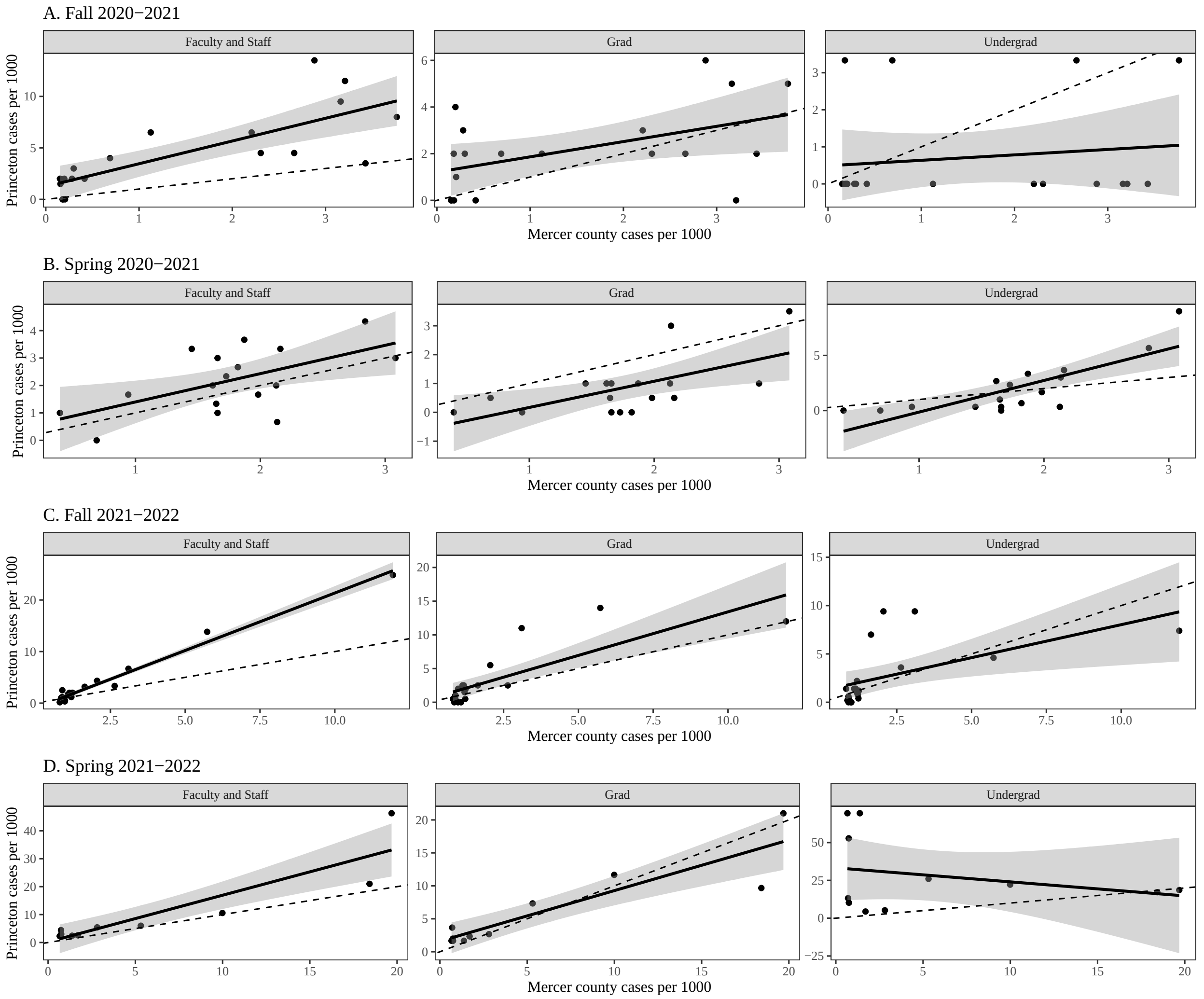

Figure S3: **Correlations between the weekly number of cases in PU and in Mercer County stratified by subpopulations.** Points represent the number of reported cases. Solid lines and shaded areas represent the regression line and the associated 95% confidence intervals.

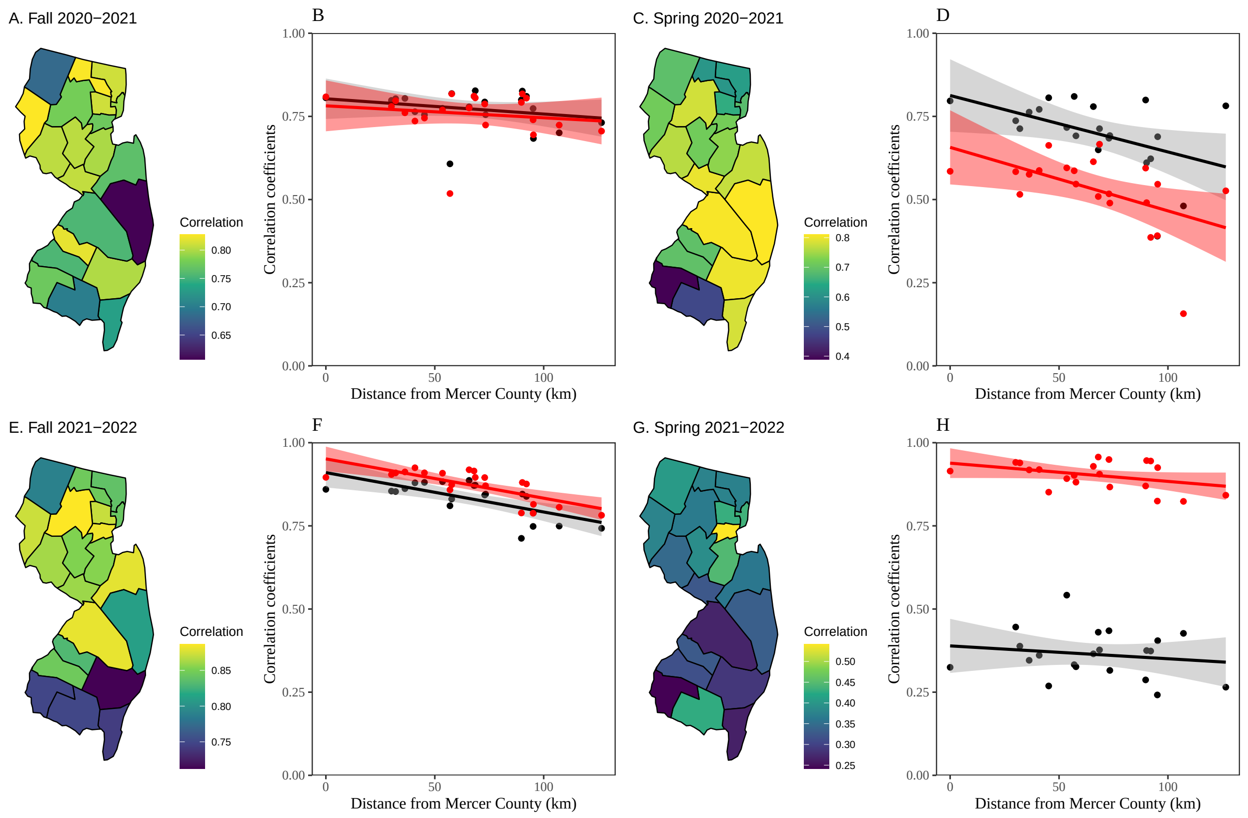

Figure S4: **Correlations between the weekly number of cases in PU and in counties in New Jersey.** (A–C) Map of correlations between the weekly number of cases in PU and in counties in New Jersey. (D–F) Relationship between case correlations and distance from Mercer County. Points represent the estimated correlation coefficients. Solid lines and shaded areas represent the regression line and the associated 95% confidence intervals. Black points and lines represent correlations based on all cases in PU. Red points and lines represent correlations based on cases among faculty and staff members in PU.

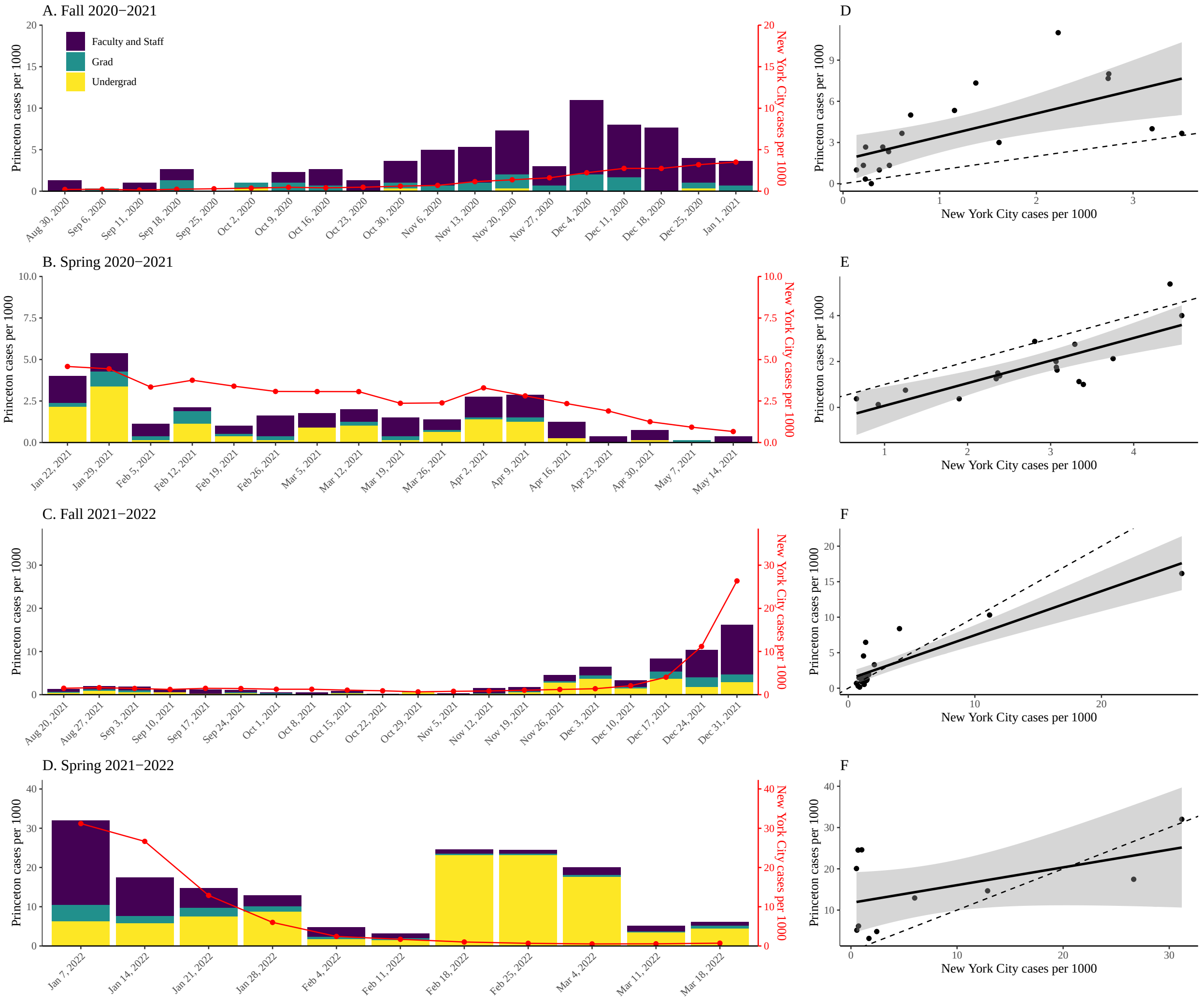

Figure S5: **Dynamics of SARS-CoV-2 outbreaks in PU and New York City.** (A–C) Epidemic trajectories across three semesters: Fall 2020 (A), Spring 2020 (B), and Fall 20201 (C). Colored bar plots represent the weekly number of cases from both asymptomatic and symptomatic testing in PU. Red lines represent the weekly number of cases in New York City. (D–F) Correlations between the weekly number of cases in PU and in New York City. Solid lines and shaded areas represent the estimated linear regression lines and the associated 95% CIs.

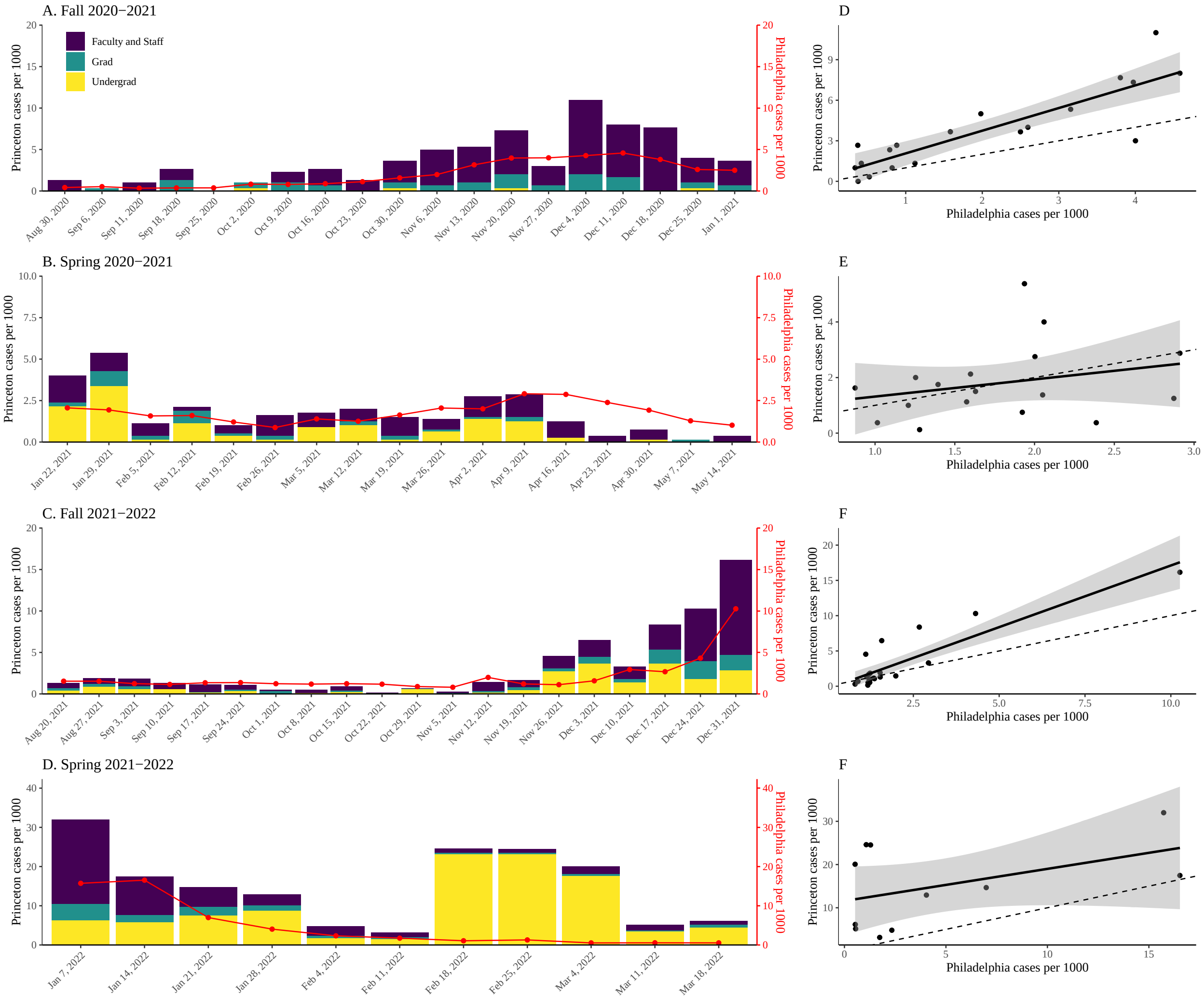

Figure S6: **Dynamics of SARS-CoV-2 outbreaks in PU and Philadelphia.** (A–C) Epidemic trajectories across three semesters: Fall 2020 (A), Spring 2020 (B), and Fall 20201 (C). Colored bar plots represent the weekly number of cases from both asymptomatic and symptomatic testing in PU. Red lines represent the weekly number of cases in Philadelphia. (D–F) Correlations between the weekly number of cases in PU and in Philadelphia. Solid lines and shaded areas represent the estimated linear regression lines and the associated 95% CIs.

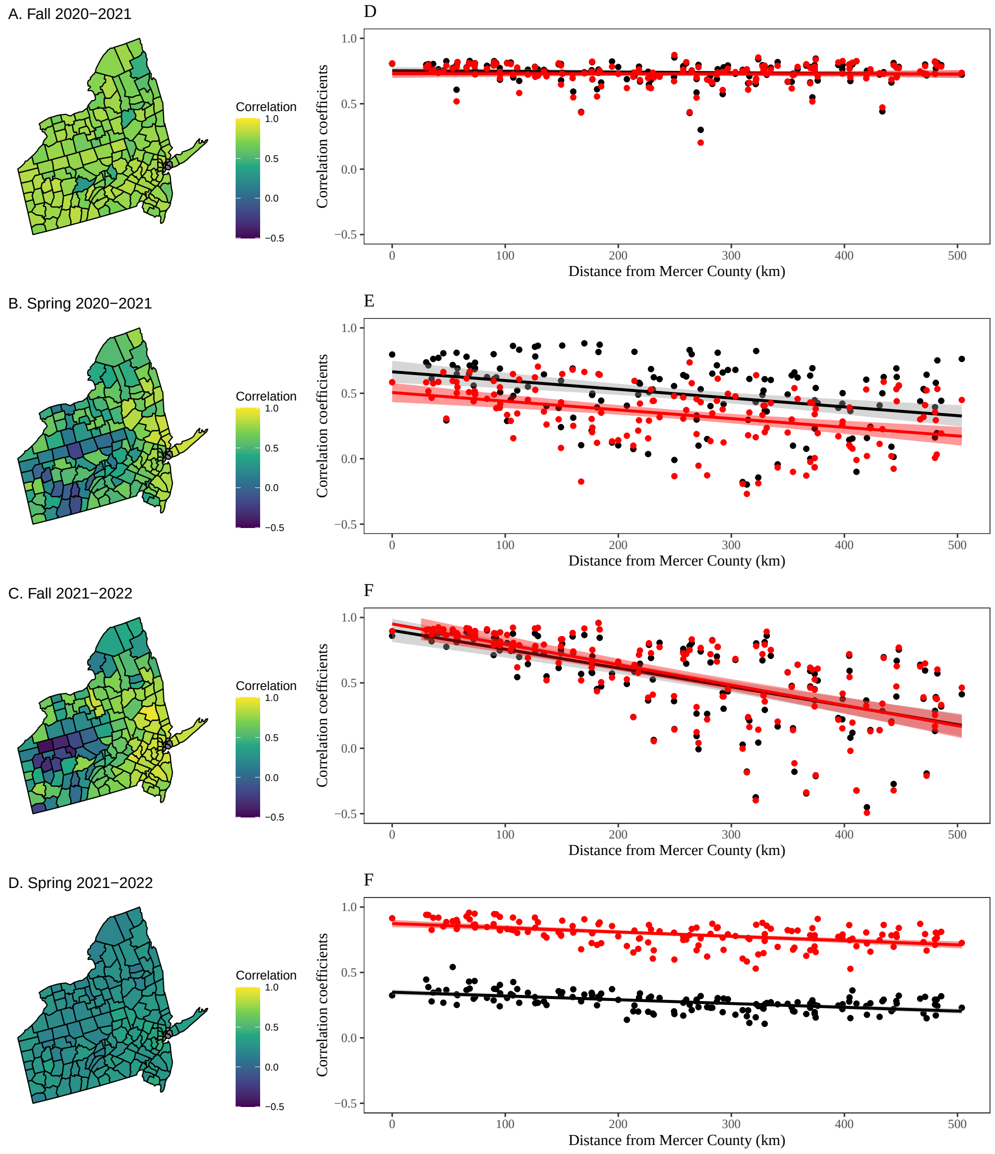

Figure S7: **Correlations between the weekly number of cases in PU and in counties in New Jersey, Pennsylvania, and New York State.** (A–C) Map of correlations between the weekly number of cases in PU and in counties in New Jersey, Pennsylvania, and New York State. (D–F) Relationship between case correlations and distance from Mercer County. Points represent the estimated correlation coefficients. Solid lines and shaded areas represent the regression line and the associated 95% confidence intervals. Black points and lines represent correlations based on all cases in PU. Red points and lines represent correlations based on cases among faculty and staff members in PU. New York City is excluded from this analysis as the data provided by New York Times are not further stratified by county levels.

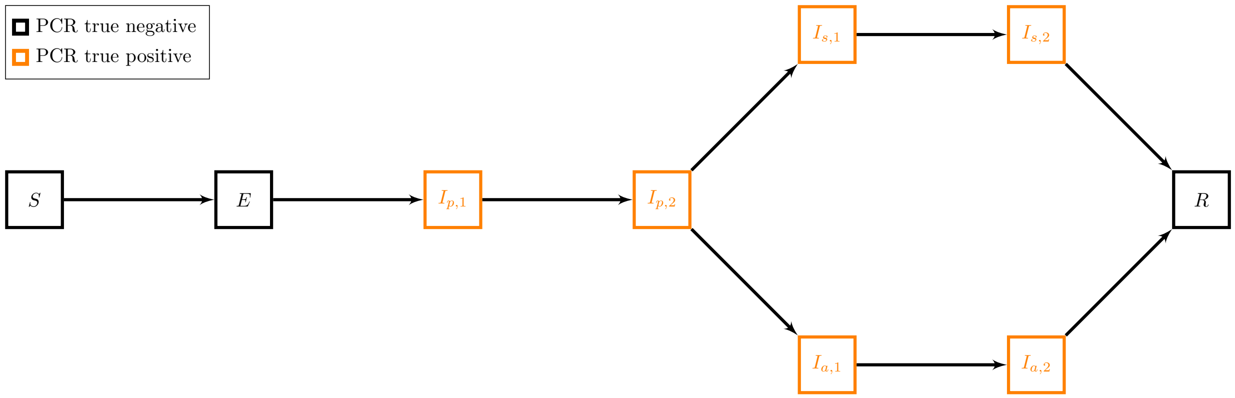

Figure S8: **Compartmental diagram of the individual-based model.** Each compartment represents a stage of infection: susceptible S, exposed S, pre-symptomatic I_p, symptomatic I_s, asymptomatic I_a, and recovered R. Pre-symptomatic, symptomatic, and asymptomatic stages are further divided into two subcompartments. Individuals in pre-symptomatic, symptomatic, and asymptomatic stages can test positive with 95% sensitivity.

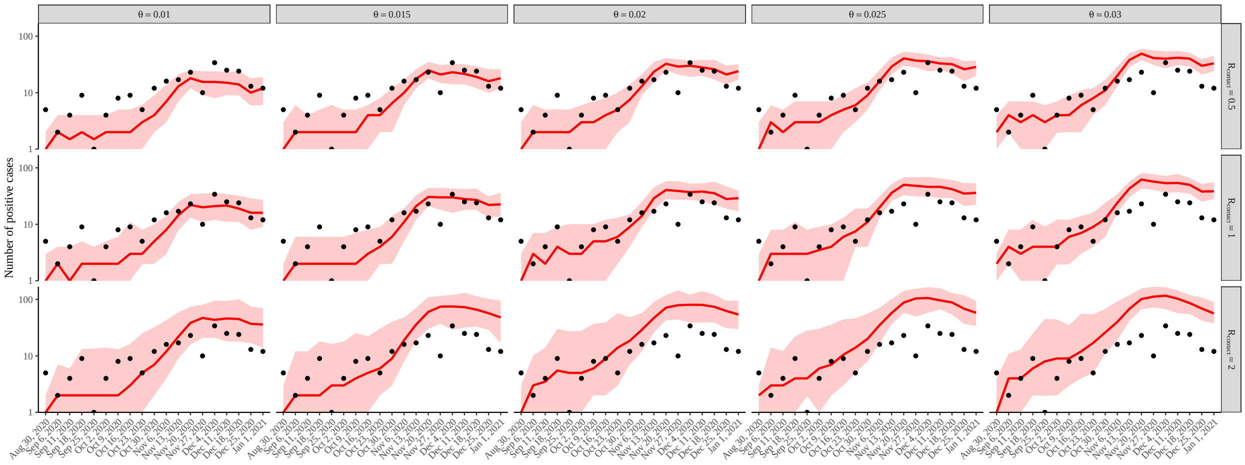

Figure S9: **Comparisons between model predictions and the observed numbers of cases for fall 2020.** Points represent the weekly number of reported cases in PU. Red lines and shaded areas represent median model predictions and 90% quantiles across 100 simulations. See figure 2 in the main text for details.

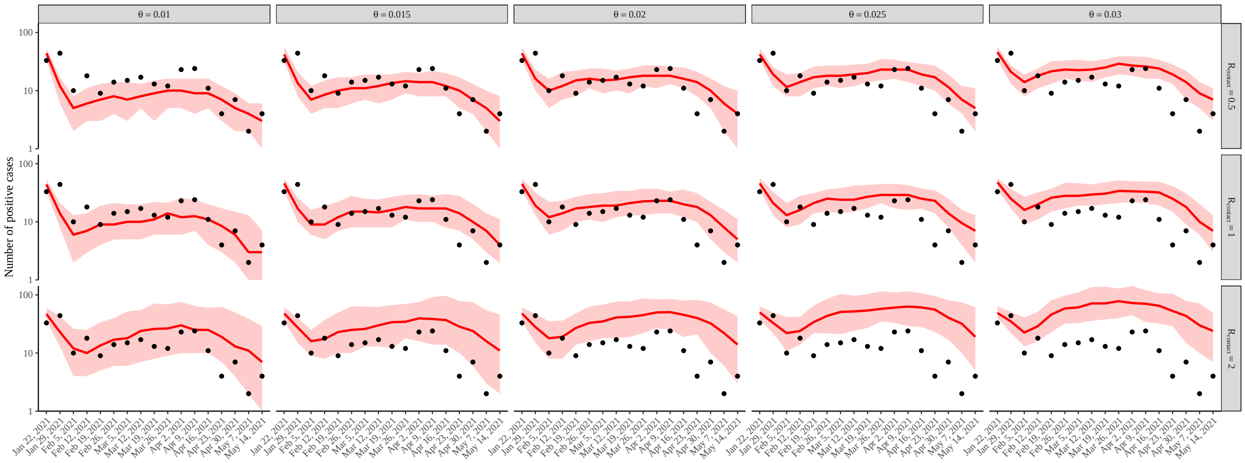

Figure S10: **Comparisons between model predictions and the observed numbers of cases for spring 2020.** Points represent the weekly number of reported cases in PU. Red lines and shaded areas represent median model predictions and 90% quantiles across 100 simulations. See figure 2 in the main text for details.

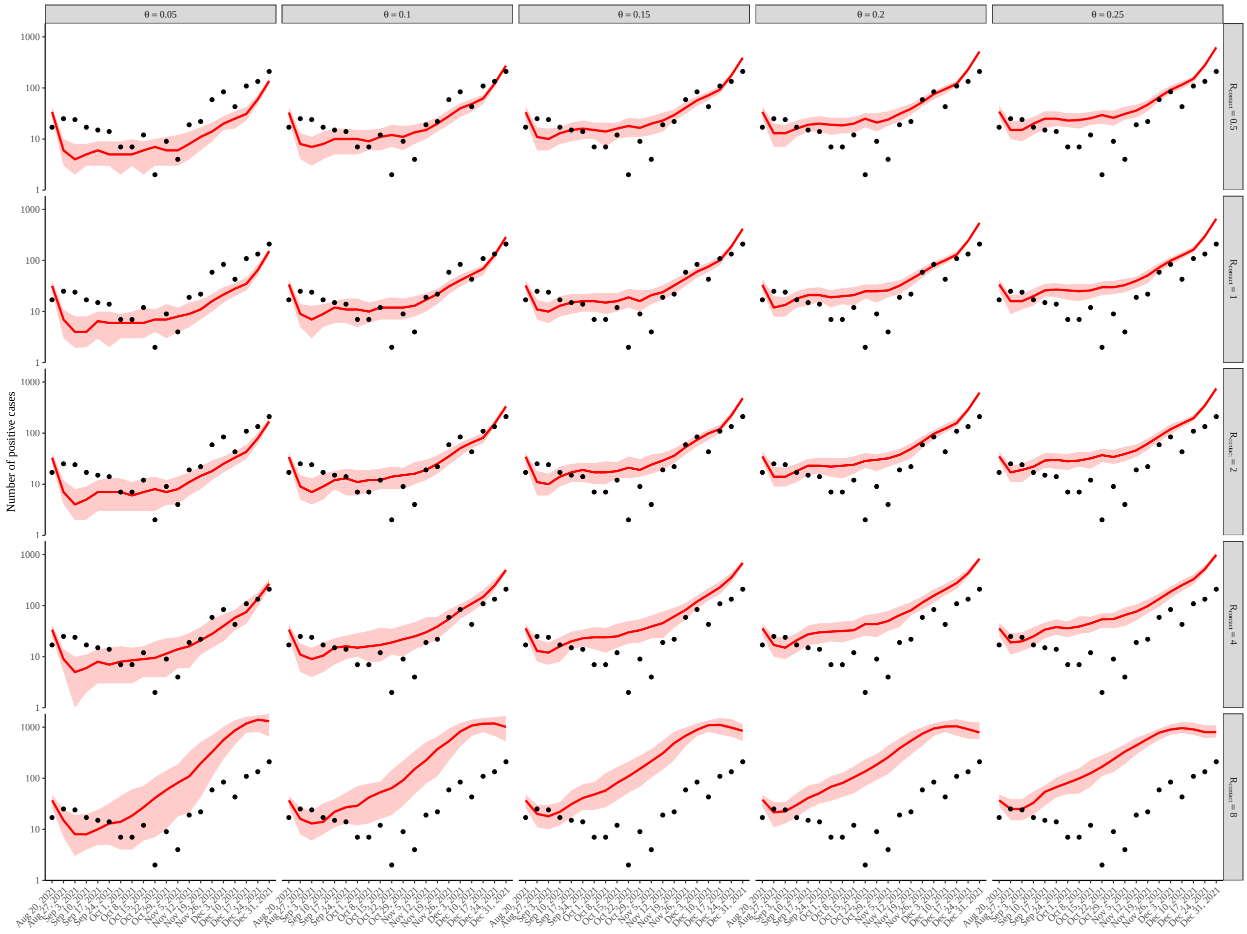

Figure S11: **Comparisons between model predictions and the observed numbers of cases for fall 2021.** Points represent the weekly number of reported cases in PU. Red lines and shaded areas represent median model predictions and 90% quantiles across 100 simulations. See figure 2 in the main text for details.

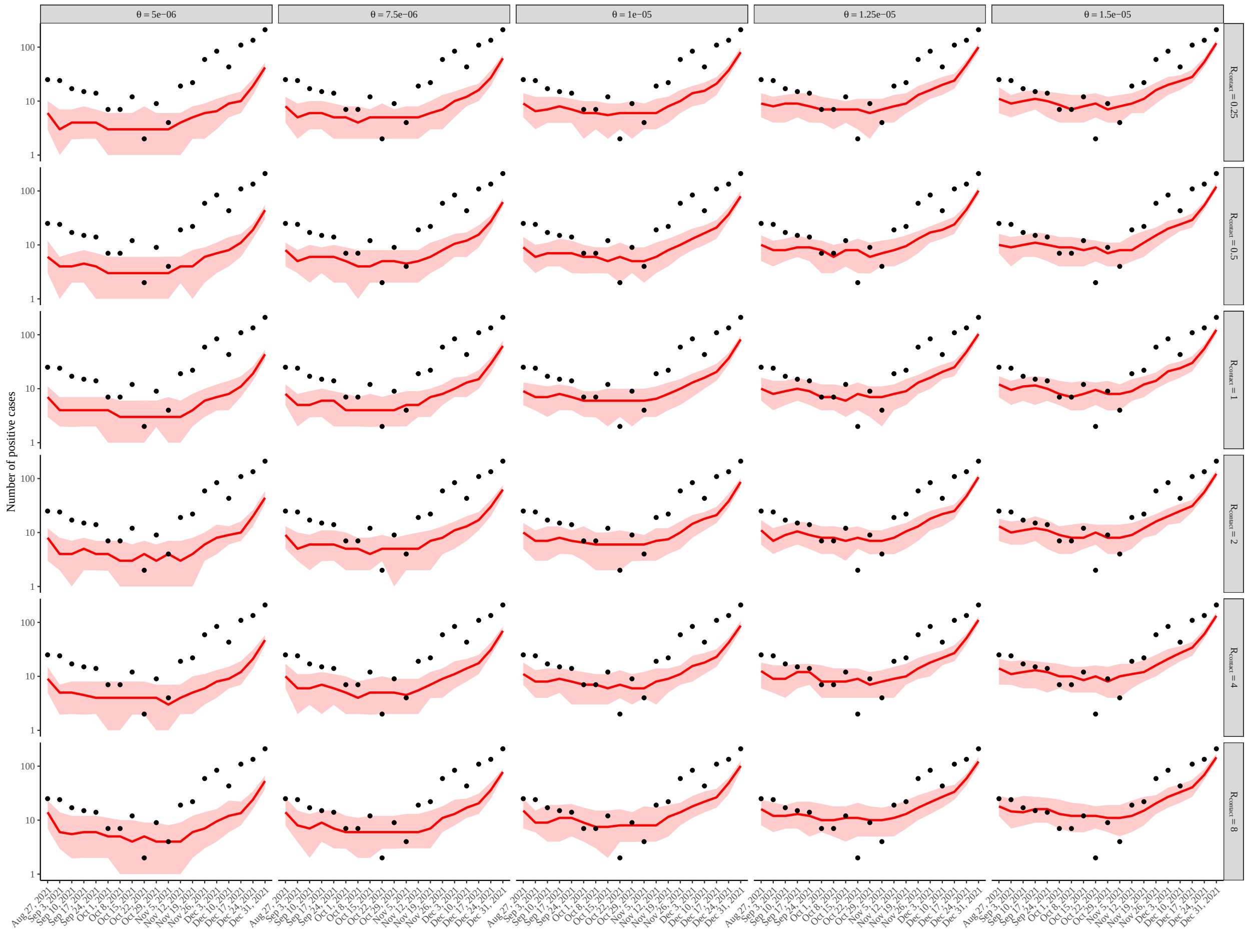

Figure S12: **Comparisons between model predictions and the observed numbers of cases for fall 2021 without immunity waning.** Points represent the weekly number of reported cases in PU. Red lines and shaded areas represent median model predictions and 90% quantiles across 100 simulations. See figure 2 in the main text for details.

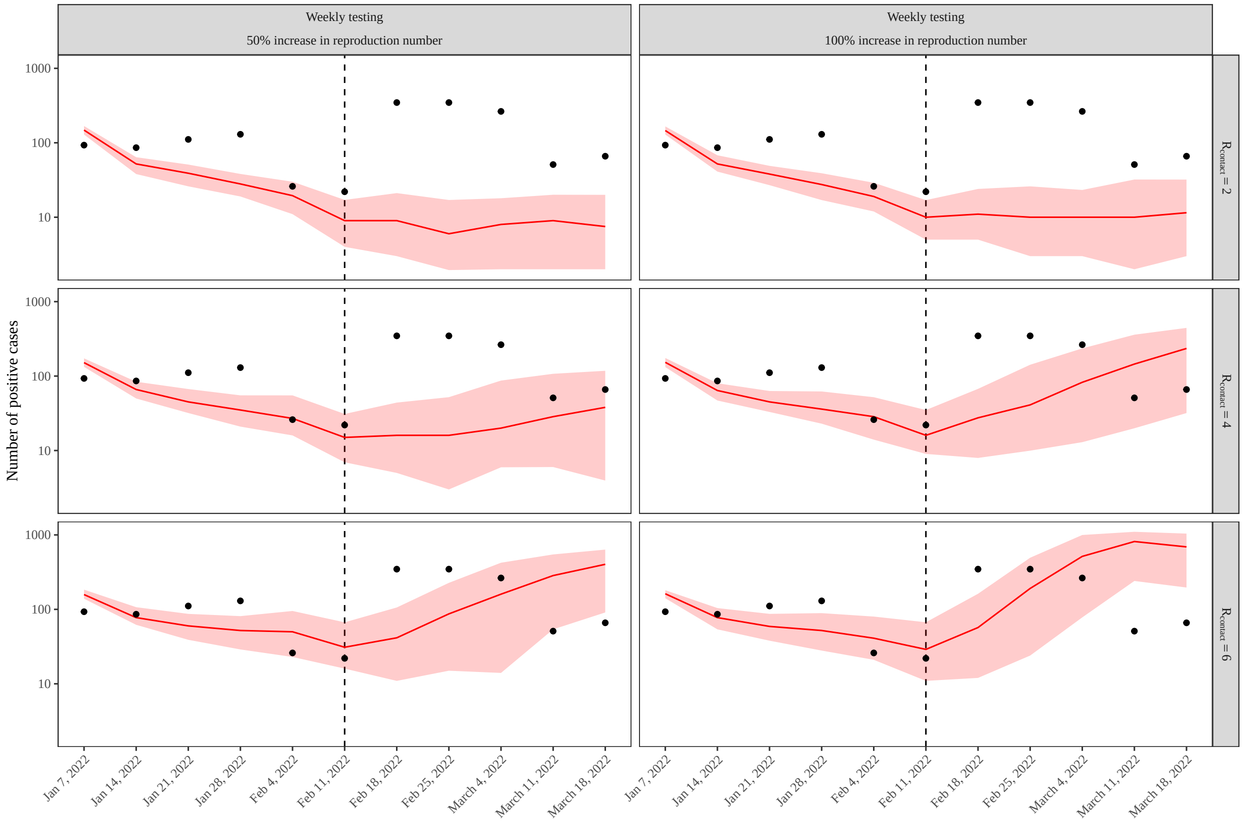

Figure S13: **The impact of changes in testing frequency and an increased reproduction number on the spread of the Omicron variant, assuming a shorter latent and infectious periods.** Solid lines represent median predictions. Shaded areas represent 90% quantiles across 100 simulations. Points represent the observed data. Vertical dashed lines represent the week including February 8, 2022, when distancing and testing policies were updated on PU campus. For each row, we assume a fixed value of baseline contact reproduction number $\mathcal{R}_{\text{contact}}$ ranging from 2 to 6 across rows. Then, we simulate increase in $\mathcal{R}_{\text{contact}}$ at the time of policy change (indicated by column labels). Here, the assumed mean latent, presymptomatic, and asymptomatic stages of infection are 0.5 days shorter than the assumed values for simulations presented in Figure 3 in the main text.

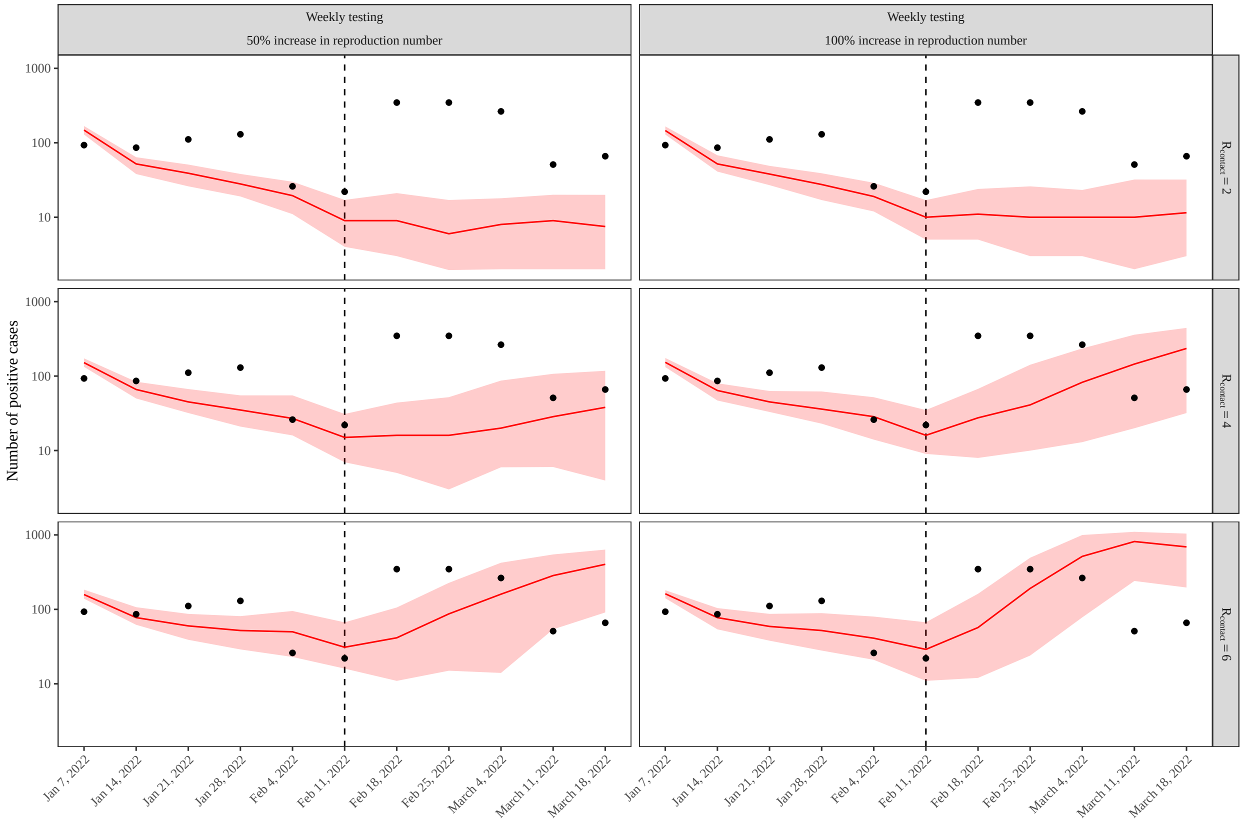

Figure S14: **The impact of changes in testing frequency and an increased reproduction number on the spread of the Omicron variant, assuming a lower vaccine effectiveness.** Solid lines represent median predictions. Shaded areas represent 90% quantiles across 100 simulations. Points represent the observed data. Vertical dashed lines represent the week including February 8, 2022, when distancing and testing policies were updated on PU campus. For each row, we assume a fixed value of baseline contact reproduction number $\mathcal{R}_{\text{contact}}$ ranging from 2 to 6 across rows. Then, we simulate increase in $\mathcal{R}_{\text{contact}}$ at the time of policy change (indicated by column labels). Here, we assume 30% vaccine effectiveness against infection.
